# Serological evidence of inter-epizootic/inter-epidemic circulation of Rift Valley fever virus in domestic cattle in Kyela and Morogoro, Tanzania

**DOI:** 10.1371/journal.pntd.0006931

**Published:** 2018-11-12

**Authors:** Mirende Kichuki Matiko, Linda Peniel Salekwa, Christopher Jacob Kasanga, Sharadhuli Idd Kimera, Magnus Evander, Wambura Philemon Nyangi

**Affiliations:** 1 Department of Veterinary Surgery and Theriogenology, College of Veterinary Medicine and Biomedical Sciences, Sokoine University of Agriculture, Morogoro, Tanzania; 2 Department of Veterinary Microbiology, Parasitology and Biotechnology, College of Veterinary Medicine and Biomedical Sciences, Sokoine University of Agriculture, Morogoro, Tanzania; 3 Department of Veterinary Medicine and Public Health, College of Veterinary Medicine and Biomedical Sciences, Sokoine University of Agriculture, Morogoro, Tanzania; 4 Department of Clinical Microbiology, Division of Virology, Umeå University, Umeå, Sweden; US Army Medical Research Institute of Infectious Diseases, UNITED STATES

## Abstract

**Background:**

Tanzania is among the Rift Valley fever (RVF) epizootic/endemic countries in sub Saharan Africa, where RVF disease outbreaks occur within a range of 3 to 17-year intervals. Detection of Rift Valley fever virus (RVFV) antibodies in animals in regions with no previous history of outbreaks raises the question of whether the disease is overlooked due to lack-of effective surveillance systems, or if there are strains of RVFV with low pathogenicity. Furthermore, which vertebrate hosts are involved in the inter-epidemic and inter-epizootic maintenance of RVFV? In our study region, the Kyela and Morogoro districts in Tanzania, no previous RVF outbreaks have been reported.

**Methodology:**

The study was conducted from June 2014 to October 2015 in the Kyela and Morogoro districts, Tanzania. Samples (n = 356) were retrieved from both the local breed of zebu cattle (*Bos indicus*) and *Bos indicus*/*Bos Taurus* cross breed. RVFV antibodies were analyzed by two enzyme-linked immunosorbent assay (ELISA) approaches. Initially, samples were analyzed by a RVFV multi-species competition ELISA (cELISA), which detected both RVFV IgG and IgM antibodies. All serum samples that were positive with the cELISA method were specifically analysed for the presence of RVFV IgM antibodies to trace recent infection. A plaque reduction neutralization assay (PRNT_80_) was performed to determine presence of RVFV neutralizing antibodies in all cELISA positive samples.

**Findings:**

Overall RVFV seroprevalence rate in cattle by cELISA in both districts was 29.2% (104 of 356) with seroprevalence rates of 33% (47/147) in the Kyela district and 27% (57/209) in the Morogoro district. In total, 8.4% (30/356) of all cattle sampled had RVFV IgM antibodies, indicating current disease transmission. When segregated by districts, the IgM antibody seroprevalence was 2.0% (3/147) and 12.9% (27/209) in Kyela and Morogoro districts respectively. When the 104 cELISA positive samples were analyzed by PRNT_80_ to confirm that RVFV-specific antibodies were present, the majority (89%, 93/104) had RVFV neutralising antibodies.

**Conclusion:**

The results provided evidence of widespread prevalence of RVFV antibody among cattle during an inter-epizootic/inter-epidemic period in Tanzania in regions with no previous history of outbreaks. There is a need for further investigations of RVFV maintenance and transmission in vertebrates and vectors during the long inter-epizootic/inter-epidemic periods.

## Author summary

The RVFV maintenance between inter-epizootic/inter-epidemic periods is not fully understood, despite the widely hypothesized belief of maintenance via transovarially infected *Aedes* mosquito eggs. Increasing serological evidence however, suggests that there could be continuous virus circulation throughout these periods in domestic ruminants, wild animals and humans both in areas with and without known history of RVF outbreaks. In some countries, RVFV antibodies have been demonstrated in livestock raised in areas where no clinical disease has ever been reported. However, in Tanzania, RVFV antibodies in livestock have been demonstrated only in areas with history of RVF outbreaks, raising the question of whether the disease is not present, is overlooked due to lack of effective surveillance systems, or whether there are strains of RVFV with low pathogenicity that do not cause detectable clinical cases in non-outbreak areas. We report here inter-epizootic/inter-epidemic RVFV antibody prevalence in non-vaccinated cattle from areas with no previous RVF outbreak in Tanzania and demonstrate recent virus circulation by detection of IgM antibodies. The differences in RVFV seroprevalence in different study locations suggest local factors that favour the virus amplification and transmission within those areas.

## Introduction

Rift Valley fever (RVF) is a zoonotic disease that causes storm abortions in ruminants [1–3]. The disease leads to introduction of restrictions for international livestock trade from enzootic/endemic countries. The disease imposes a dual impact in that it exacerbates the poverty cycle in livestock-dependent communities, by causing substantial health costs and at the same time affecting negatively the livelihoods of the communities in many sub-Saharan countries where it is enzootic/endemic[4,5]. RVF was first reported in early 1930’s in the Eastern Rift Valley province of Kenya causing high rates of abortion in infected sheep [6]. Since then, the Rift Valley fever virus (RVFV) has been associated with several periodic disease epidemics and epizootics affecting human and animals in many regions of Africa. Although the virus is enzootic/endemic to sub-Saharan Africa, it has the potential for global spread and has already crossed significant natural geographic barriers such as the Indian Ocean, the Sahara Desert and the Red Sea to reach naive ecologies [7]. Outside Africa, RVF outbreaks were first reported in Saudi Arabia [8] and Yemen [9] in 2000. This northward spread of RVFV suggests the possibility of the virus being introduced into Europe and North America where several species of mosquitoes competent for viral transmission exist [10].

Recent spatial and temporal analysis of RVF in Tanzania showed that RVF-like disease was reported for the first time in 1930 concurrently with the outbreak in Kenya, with a further ten outbreaks being reported between 1947 and 2007 [7]. In 2006/2007, there was a massive outbreak with a total of 684 human cases and 234 deaths reported in Kenya, 114 cases with 51 deaths in Somalia and 264 cases with 109 deaths reported in Tanzania [11]. In Tanzania, the 2006/2007 RVF outbreak was widely spread to more than ten regions in the northern, eastern-central and southern parts of the country [7].

In RVFV enzootic/endemic regions, outbreaks occur with 3 to 17 year intervals [12], which is an average inter-epizootic/inter-epidemic (IE) interval of 7.9 years [7]. The RVFV maintenance between the long IE periods is not fully understood. Although it has been widely hypothesized that the virus is maintained via transovarially infected *Aedes* mosquito eggs [12,13], serological evidence suggests that the virus could be maintained through IE circulation in domestic ruminants, wild animals and humans [9,14–17]

Evidence of RVFV circulating in Tanzania during an IE period has been shown previously in the Kilombero river valley, where various livestock species had RVFV antibodies (cattle 11.03%; sheep 11.86% and goats 11.37%, respectively) [14], although Kilombero river valley was among the sites that experienced the 2006/07 RVF outbreak [7]. Additional surveys in areas without previous history of RVF outbreaks [18] or clinical cases in humans [9] show related prevalence rates in livestock and humans respectively. In addition, the role of mammals as maintenance hosts for RVFV remains largely unknown [19]. Furthermore, the detection of antibodies in areas where no clinical disease has been reported [9,18] raises the question of whether the disease is overlooked due to lack-of effective surveillance systems, or whether there are strains of RVFV with low pathogenicity. This study aimed at determining the involvement of non-vaccinated cattle in the IE maintenance and transmission of RVFV in areas with no history of RVF outbreaks in Tanzania.

## Materials and methods

### Study areas

This study was conducted from June, 2014 to October, 2015 in the Kyela and Morogoro districts, Tanzania. During the 2006/2007 RVF outbreak in Tanzania, ten regions were affected [20]. The Morogoro region was one of the ten regions, but only two districts, the Kilombero and Ulanga districts were affected, while the Morogoro district was not affected [7]. The Mbeya region, where the Kyela district is located, was not affected during the 2006/2007 RVF outbreak in Tanzania [7,20].

Kyela is one of the districts in Mbeya region, located in south-western part of the country. Most of the Kyela district is lowland situated in the Great Rift Valley at 505m above sea level, in the flood plains of Lake Nyasa. It receives heavy rains, of about 2000-3000mm per annum and floods are common in March through May. The district has a warm and humid climate, with a mean daily temperature of 23°C. Together with Lake Nyasa, the district also has four large rivers, (Songwe, Mbaka, Lufilyo, and Kiwira), and many streams (Mkalizi, Kampala, Mgaya, Chiji, Kandete, Masukila, Njisi, and Kubanga). Agriculture dominates livelihoods and economic activities of the Kyela district. In addition to rain fed paddy farming, other crops include banana and cocoa cultivation. Other livelihood activities include livestock farming and fishing. Because of the water logging condition, few sheep and goats are kept in the district. Few (1–5) cattle are kept per household by tethering in communal grazing areas during the day and on the doorsteps of their houses at night for fear of theft, providing an animal reservoir of RVFV in proximity of humans.

The Morogoro district is located within the Morogoro region, 200 km east of Dar es Salaam. The annual average rainfall for Morogoro ranges between 500 and 1800 mm with temperatures between 18°C to 28°C. The main occupation of the inhabitants include crop cultivation and livestock keeping and a number of livestock species are kept including cattle, goats, sheep, pigs, camels, donkeys and horses. The livestock production in Morogoro is organised under commercial and traditional sectors. The livestock production systems are pastoralisim, agro-pastoralism and small scale intensive system which is becoming popular as land shortage force many livestock keepers to intensify their production. In the latter system, mainly crossbred animals are kept, and cut and carry system of feeding is practicied.

### Selection of districts and wards

The sampling process involved a two-stage purposive selection of districts and wards based on the findings of the past studies (2) reporting status of RVF outbreaks in Tanzania. The number of wards was not based on statistical considerations, but on logistic and resource availability.

Based on the above, the Kyela and Morogoro districts with no previous history of RVF outbreaks were selected. In both districts, all veterinary officers were consulted to identify wards within each district considered to be at highest risk of RVF occurrence. Criteria used included areas subject to regular flooding, ecological features suitable for mosquito breeding, relatively high concentration of domestic ruminants, proximity to rivers, ponds and lakes. The wards within the districts that were identified with most of these epidemiological characteristics were selected for the study.

Within the selected wards, all households keeping domestic ruminants and not having a history of vaccination against RVF were identified using local official veterinary records. The spatial and temporal patterns of RVF outbreaks in Tanzania; 1930 to 2007 [7], showed that no previous outbreak had occurred in the two study districts. It was further confirmed during the study where, in each district, the veterinary offices were asked for any occurrence of RVF disease and/or outbreak, and history of livestock RVFV vaccination. Furthermore, information on the animal movements into the selected wards was retrieved from the veterinary officials, household heads and herdsmen. Additional information on the sources of replacement heifers was also requested. Wards without inward migration of animals from other areas were selected for the study.

### Sample collection

The local breed of zebu cattle (*Bos indicus*) and the crosses with exotic breed (*Bos taurus*) were sampled by collecting 5 ml of blood from the jugular vein into plain vacuitaner tubes. The criteria for selection of animals included a history of non-vaccinated status against RVFV, animals born after the 2006/2007 outbreak, calves above 6 months of age, and owners consent to using the animals for study. Herd and individual animal epidemiological data were obtained from the household head and herders as well as through clinical examination. The data collected included the breed, sex and age and feeding practices. In addition, a history of animal movements into the herd and whether the animals were born within the herd or introduced (moved) into the herd from another district was recorded. Sampling was based on only those herds with restricted animals without history of movements to high RVF risk areas.

The blood samples were collected in vacutainer tubes without an anti-coagulant, labeled and stored in a cooler box with ice packs while in the field. Before blood collection, animals were restrained into the crush or by use of ropes and halters. The blood was allowed to coagulate before serum was separated into a 1.5 ml cryovial tube, labeled and stored in a cool box with ice packs until transfer to the laboratory for analysis. Serum samples were stored at -80°C until analysis.

### Age and breed determination

Individual animal age was estimated from epidemiological data collected from household heads and herders, and where possible, by review of available records on date of birth and dentition. Records were available from farms keeping crossbred dairy cattle. Dentition was used in determining the age of cattle divided into young or adult, depending on the eruption of the permanent incisors [21]. All cattle that had at least a permanent middle incisor were categorised as adult, while those without were categorised as young. Normally, the permanent incisors in cattle erupt at about 18 months of age and by 24 months they are fully developed. To exclude sampling young animals less than six months old, age was estimated by asking the head of the household, herd boys and other members of the household for the month, season and year of birth. Also, we performed physical observation of animal size and asked if they still were suckling. To avoid sampling animals present during the 2006/2007 RVF outbreak, animals that had initial wear on their incisor teeth (5 to 6 years old) and those which had noticeable wear (7 to 8 years old) were excluded from the study.

Breed types were recorded as local or cross-breeds, depending on the body colouration, presence of a hump and horns. Local breeds were shorthorn humped zebu with various body colouration. Sex of the animal and test results were provided in the same data set.

### Multi-species competition enzyme linked immunosorbent assay (cELISA)

Each serum sample was analysed with the commercial Innovative Diagnostic (ID.vet) Screen RVF competition multispecies ELISA (cELISA) (IDVet, Montpelier, France). The commercial cELISA is based on the recombinant RVFV nucleoprotein and detects both RVFV IgM and IgG antibodies. The cELISA was carried out according to the manufacturer’s instruction. Briefly, 50 μl of the dilution buffer was dispensed into each well of a labelled ELISA plate pre-coated with recombinant RVFV nucleoprotein. Then, 50 μl of the internal positive (freeze-dried RVFV IgG positive bovine serum supplied by the manufacturer) and the internal negative control (supplied by the manufacturer) were added in duplicates. To the remaining wells, 50 μl of each sample was added. After mixing samples and controls with the TST dilution buffer (50 mM Tris/150 mM NaCl/0.1% Tween 20, pH 8.0), we incubated at 37°C for 1 hour. The wells were washed three times with washing buffer using a well plate washer (Thermo Scientific Wellwash Microplate Washer, Waltham, MA USA). Next, 100 μl of antinucleoprotein peroxidase (HRP) conjugate was added to the wells and the contents of the plate were incubated at room temperature for 30 min, followed by washing three times with 300 μl of wash solution as before to remove excess conjugate. Then, 100 μl of substrate solution 3,3',5,5'-tetramethylbenzidine (TMB) was added to each well and the plate was incubated at room temperature for 15 min in the dark. To terminate the reaction 100 μl of 2n Sulphuric acid (2NH_2_SO_4_) stop solution was added to each well. The presence of antibodies to RVFV was detected by lack of a colour change, whereas absence of antibodies to RVFV was detected by a change in substrate colour to blue. The contents of the wells of the microplate were read at a wavelength of 450 nm by a microplate absorbance reader (Molecular Devices, CA, USA).

For each cELISA experiment, duplicate internal controls were incorporated. The optical densities (ODs) of the control were detected at 450 nm. To verify the reliability and validity of the results obtained from each cELISA test, the average of the ODs of the two negative controls (NCs) was > 0.7 while the average of the two positive controls divided by the average OD of the NCs was > 0.3. For each sample, the competition percentage was calculated by dividing the OD of the sample by the average OD of the negative control multiplied by 100 ([ODsample/ODNC] x 100). A sample was considered positive if the value obtained from the formula was ≤ 40%. Any sample with a value of > 50% was considered to be negative, whereas values ranging from 40–50% were considered to be doubtful.

### RVFV IgM antibody capture ELISA

The IgM ELISA test was employed for cELISA positive samples only. These samples were analysed with the commercial ID Screen RVF IgM Capture kit (IDvet, MOntpelier, France) according to the manufacturer’s instruction. Briefly, 40 μl of the diluent buffer was dispensed into each well of a labelled microwell plate pre-coated with anti-bovine-ovine-caprine IgM polyclonal antibodies. Then, 10 μl of the internal positive control (freeze-dried anti-RVFV recombinant NP bovine serum supplied by the manufacturer) and the internal negative control (supplied by the manufacturer) were added in duplicates. To the remaining wells, serum samples were added in duplicate and the plate with all samples was incubated at 37°C for 1 hour. The microplate wells were then washed three times with 300 μl by a microplate washer as above. Next, 50 μl of RVFV nucleoprotein or diluent buffer was added and incubated at 37°C for 1 hour. The wells were washed three times followed by addition of 50 μl of anti-RVFV nucleoprotein horseradish peroxidase (HRP) conjugate solution to each well and incubation for 1 hour at 37°C. Again, the wells were washed three times as above and 100 μl of the substrate solution, TMB, was added to each well and then incubated for 15 min at room temperature in the dark. Then, 100 μl of stop solution was added to terminate the reaction.

The presence of IgM antibodies to RVFV was detected by appearance of blue colouration, which became yellow after addition of the stop solution. The contents of the wells of the microplate were analysed at 450 nm by a microplate absorbance reader (Molecular Devices, CA, USA).

For each IgM antibody capture ELISA experiment duplicate internal controls were incorporated. The optical densities (ODs) obtained from the samples at 450 nm were validated in accordance with the manufacturer’s instructions as follows:

The net OD was calculated: net OD = OD _even well_-OD _odd well_

The plate was valid if the mean value of the net positive control OD was greater than 0.35 and the ratio of the mean values of the net positive and negative control (absolute value of ODs) is greater than 3 (net OD_PC_/net OD_NC > 3_

### Interpretation of antibody detection results

For each sample, the percentage of the ratio of sample and positive control (s/p%) was calculated.

$${S/P\%=\mathrm{net}\;\mathrm{OD}}_{\mathrm{sample}}{/\mathrm{net}\;\mathrm{OD}}_{\mathrm{postive}\;\mathrm{control}}$$

Samples presenting a S/P percentage (S/P%):

1. Less than or equal to 40% were negative
2. Between 40% and 50% were doubtful
3. Greater than or equal to 50% were positive

### Plaque reduction neutralization test 80% (PRNT_80_)

All samples that were positive for RVFV antibodies by the cELISA kit were analyzed by PRNT_80_. The PRNT_80_ protocol used was adopted as previously described [22]. The RVFV MP-12 vaccine strain, propagated in Vero-E6 cells, was used in the PRNT assay.

Each PRNT assay included the test sera, and a known RVFV antibody positive serum sample and a RVFV antibody negative serum sample from cattle. Each serum sample was diluted in Hanks’ Balanced Salt Solution (HBSS) supplemented with one % each of HEPES, penicillin and streptomycin and heat-inactivated fetal bovine serum (FBS). The dilutions sera samples were made in 96 well plates beginning with a 1:5 dilution in the first wells followed by 4-fold serial dilutions of 1:20, 1:80, 1:320, 1:1280, and 1:5120 in each of subsequent wells. Each diluted serum sample was then mixed with an equal volume of 60–80 plaque-forming units (PFU) of MP-12 vaccine virus. The quantification of PFU was confirmed by a plaque assay based on testing a mixture of equal volumes of the 60–80 PFU and HBSS to confirm that the final virus dose ranged from 30–40 PFUs. The antibody positive control consisted of a mixture of equal volume of 60–80 PFU and a 1:10 dilution of antibody positive cattle serum. The antibody negative control consisted of a mixture of equal volume of 60–80 PFU and a 1:10 dilution of RVFV antibody negative cattle serum. The virus/serum dilution mixtures were incubated at 37°C in the absence of CO_2_ for one hour. Next, 50 μl of the virus/serum dilution mixtures were inoculated onto each of two Vero E6 cell monolayer cultures propagated in 24-well tissue culture plates and incubated for one hour at 37°C and 5% CO_2_. Virus mixed with the antibody-positive control serum, was inoculated onto twenty separate Vero E6 cultures. Virus mixed with antibody-negative control serum mixture was inoculated onto four Vero E6 cultures. After incubation for one hour at 37°C with 5% CO_2,_ each cell culture was overlaid with 0.5 ml of a Seakem agarose (1%) with an equal volume of 2X Eagle’s Basal Medium with Earle’s salts (EBME) supplemented with 8% FBS and one % penicillin/streptomycin, and Glutamine+8g/l HEPES. After two more days of incubation at 37°C with 5% CO_2_, each culture was overlaid with 0.5 ml of a mixture of an equal volume of agarose (1%) and 2X EBME supplemented with 5% neutral red, 8% FBS, and penicillin and streptomycin (1%) and Glutamine + 8g/l HEPES and incubated overnight at 37°C with 5% CO_2_. The PFUs were counted and recorded for both the controls and cattle serum samples. An 80% reduction in the number of PFUs was used as the endpoint for antibody virus-neutralization titers (PRNT80). Wells with too high number of PFUs, that were impossible to count at that dilution, were recorded as TNTC (too numerous to count).

### Data analysis

The data were entered into a Microsoft Excel spreadsheet and imported into STATA version 12 (Statacorp, College Station, TX, USA) for cleaning and statistical analysis. Descriptive statistics was carried out followed by univariable analysis to assess initial association between potential risk factors and the outcome variable defined by RVFV seropositivity. The mixed effects logistic regression modelling was used to investigate the association between various potential risk factors and the outcome variable defined by RVFV seropositivity. The models included districts, age, breed, sex and the type of holding. The analysis was conducted in two steps. The statistically significant variables were included in a mixed effects multivariable logistic regression analysis based on a forward variable selection approach, utilising the likelihood ratio statistic and a *p*-value ≤ 0.05. Because of the differences in the sample sizes and agro-ecological features between Kyela and Morogoro districts, RVFV seropositivity was compared among the wards within the respective district. The Chi-square test was used to compare the RVFV seropositivity among the wards, by using the Rstudio statistical software at *p*-value ≤ 0.05.

### Ethics statement

During blood collection from cattle, the research team adhered to the generally acceptable ethical standards and strictly followed existing national and international guidelines for minimizing pain and stress to the animals. The study purpose was explained to cattle owners prior to sample collection and upon agreeing to allow samples to be collected from their animals, they provided a written consent form. The study protocol was approved by Research and Publication Committee, College of Veterinary and Biomedical Sciences, Sokoine University of Agriculture, Tanzania.

The protocol/permit number assigned by the Institutional Animal Care and Use Committee IACUC/ethics committee Protocol No. SUA/FVM/R.19 of 17^th^ March 2014.

National or international regulations/guidelines to which animal care and use protocol adhered to: Public health Service Policy on Humane Care and Use of Laboratory Animals and Animal Welfare Regulations.

## Results

A total of 356 cattle serum samples were analysed for presence of RVFV antibodies, of which 147 samples were from the Kyela district and 209 samples were from the Morogoro district. The overall seropositivity by cELISA was 29.2% (104/356) and a seroprevalence of 32% (47/147) and 27% (57/209) were recorded among animals in Kyela and Morogoro districts respectively. Animals older than 2 years were more likely to be seropositive than animals younger than 2 years (OR = 0.19; *p* = 0.000) (Table 1). Likewise, zebu cattle were more likely to be seropositive than crosses, (OR = 2.5; *p* = 0.000). There were no significant differences between the districts (OR = 0.98; *p* = 1.0) and the type of holding (OR = 1.25; *p* = 0.34).

**Table 1:** Potential risk factors related to RVFV seroprevalence in cattle in two districts of Kyela and Morogoro, Tanzania.

**Variable**	**Level**	**% cELISA positive (n)**	**Odds ratio (*OR*)**	**95% Confidence Interval (CI)**	**p-value**
District	Kyela (n = 147)	32.0(47)	0.8	0.50–1.27	0.34
	Morogoro (n = 209)	27.3(57)			
Age	Adult (n = 234)	38.9(91)	0.19	0.1–0.35	<0.001*
**Variable**	**Level**	**% cELISA positive (n)**	**Odds ratio (*OR*)**	**95% Confidence Interval (CI)**	**p-value**
District	Kyela (n = 147)	32.0(47)	0.8	0.50–1.27	0.34
	Morogoro (n = 209)	27.3(57)			
Age	Adult (n = 234)	38.9(91)	0.19	0.1–0.35	<0.001*

	Young (n = 122)	10.7(13)			
Breed	Cross (n = 180)	20.0(36)	2.5	1.57–4.05	<0.001*
	Zebu (n = 176)	38.6(68)			
Sex	Female (n = 273)	29.3(80)	0.98	0.57–1.69	0.95
	Male (n = 83)	28.9(24)			
Holding	Farm (n = 209)	27.3(57)	1.25	0.79–1.99	0.34
	Open (n = 147)	32.0(47)			

*Statistical significant differences between the groups

**Fig 1:**
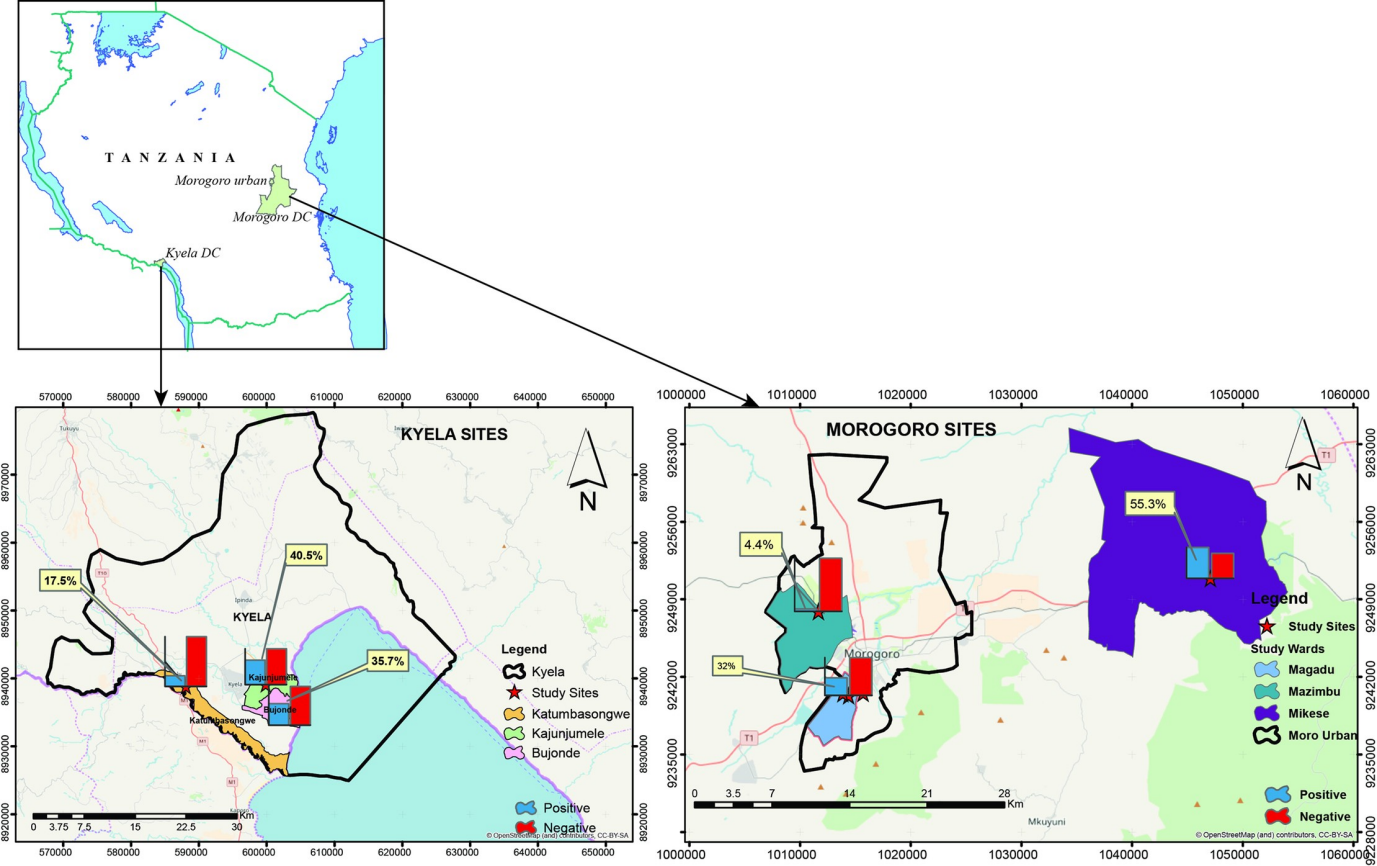
Seroprevalence of RVFV in cattle from different wards in Kyela and Morogoro districts.

In the Morogoro district, the Mikese ward had the highest RVFV seroprevalence at 55.3% (21/38) followed by Magadu at 32% (33/103) and Mazimbu 4.4% (3/68). In the Kyela district, the RVFV seroprevalence was 35.7% (25/70), 40.5% 15/37) and 17.5% (7/40) in Bujonde, Kajujumele, and Katumba Songwe wards respectively (Fig 1, Table 2). There were statistical significant differences in RVFV seroprevalence between the wards in Morogoro district (*p* < 0.001) (Table 2). However, this was not the case for wards in the Kyela district (*p* = 0.06) (Table 2).

**Table 2:** Distribution of RVFV seroprevalence in different wards in Kyela and Morogoro districts, Tanzania.

District	Ward	Samples tested (n)	cELISA positives (n)	% cELISA positive (n)	Chi-square	Df	p-value
Kyela	Bujonde	70	25	35.7	5.55	2	0.06
	Kajunjumele	37	15	40.5			
	Katumba Songwe	40	7	17.5			
Morogoro	Magadu	103	33	32.0	34.11	2	< 0.001*
	Mazimbu	68	3	4.4			
	Mikese	38	21	55.3			

*Statistical significant differences between the groups

To specifically detect RVFV IgM antibodies, the RVFV antibody positive samples analysed by the cELISA method were subjected to an IgM capture ELISA. Of the 104 analyzed samples, 30 (29%) were positive for RVFV IgM antibodies. In total 8.4% (30/356) of all cattle sampled in the two districts had RVFV IgM antibodies. When segregated by districts, the IgM antibody seroprevalence was 2.0% (3/147) and 12.9% (27/209) in Kyela and Morogoro districts respectively. In the Morogoro district, the RVFV IgM seroprevalence was 11.7% (12/103), 1.5% (1/68) and 36.8% (14/38) for Magadu, Mazimbu and Mikese wards respectively while in Kyela district, the IgM seroprevalence was 2.9% (2/70), 0% (0/37) and 2.5% (1/40) in Bujonde, Kajunjumele and Katumba Songwe wards respectively (Table 3).

**Table 3:** Distribution of RVFV IgM seropositivity and neutralizing RVFV antibodies in Kyela and Morogoro districts, Tanzania.

Region	Variable	Level	Total samples (n)	IgM positives (n)	% IgM positive	% PRNT_80_ positive of cELISA positives (n)*
Mbeya	District	Kyela	147	3	2.0	100 (n = 47)
					
	Wards	Bujonde	70	2	2.9	100 (n = 25)
		Kajunjumele	37	0	0	100 (n = 15)
		Katumba Songwe	40	1	2.5	100 (n = 7)
Morogoro	District	Morogoro	209	27	12.9	81 (n = 57)
					
	Wards	Magadu	103	12	11.7	85 (n = 33)
		Mazimbu	68	1	1.5	0 (n = 3)
		Mikese	38	14	36.8	86 (n = 21)

*The plaque reduction neutralization (PRNT) assay detected neutralizing RVFV antibodies

Positive samples by cELISA were also analyzed for presence of RVFV neutralising antibody by the PRNT_80_ assay and 89% (93/104) of all cELISA-positive samples were PRNT-positive. All (47/47) cELISA positive samples from the Kyela district contained RVFV neutralising antibody, while 81% (46/57) of the samples from Morogoro district had neutralising antibody. Antibody titres ranged from 1:10 to 1:10240 and above (S1 Table). Some ELISA positive cattle samples collected from wards in Morogoro district gave titers below 1:10 which was considered negative.

## Discussion

We found IgG and/or IgM antibodies to RVFV in 29.2% of cattle sampled during 2014–2015 in Tanzania, from two districts with no RVF outbreaks. All samples were collected during an inter-epizootic/inter-epidemic (IE) period from animals born after the large RVF outbreak in East Africa 2006/2007. The finding of both IgG and IgM positive cattle suggests both long-term persistence of RVFV antibodies and a low level of recent circulation of RVFV. In previous studies from Kilombero, Tanzania and Ijara Kenya the seroprevalence in livestock born after the 2006/2007 outbreak was only 5.5% and 13.1% respectively, while in a study in Tanzania from 2013 in the Kajunjumele ward in the Kyela region, 25.8% had RVFV IgG antibodies [14,23,24]. Interestingly, we detected a RVFV seroprevalence of 40.5% (15/37) in the Kajunjumele ward from samples collected 2014–2015, but none of the fifteen seropositive animals were RVFV IgM positive. This suggested that new infections have occurred in the Kajunjumele ward between the previous study, ending August 2013, and our study, starting June 2014. This should then have occurred at least 6–8 weeks before our sampling, since IgM antibodies only persist for that time (25), but no reported animal or human cases were reported from that region during the period. On the other hand, cattle samples collected from Tanzania during the 2006/2007 outbreak had a seroprevalence of 38.7% [25]. The variation in seroprevalence could be explained by time of sampling, new infections, slaughter, removal of seropositive animals, methods used to analyze the samples, as well as the agro-ecological conditions of the study sites.

The results reported in this study indicated that domestic cattle from the two studied districts have been exposed to RVFV infection during the IE period and could function as virus amplifiers, although the two study districts have no previous history of RVF outbreaks. The cELISA method detected both IgG and IgM RVFV specific antibodies. The RVFV IgG antibodies are believed to persist in animals for life following infection, and therefore its detection provides a reliable index of previous exposure to RVFV (5, 6), but does not indicate when the animals were infected. The detection of RVFV IgM antibodies indicated that the virus was actively circulating sub-clinically in the both the Kyela and Morogoro districts during the time of sampling, although mainly in the Morogoro district. This is supported by the fact that IgM antibodies persist for only 6 to 8 weeks after initial infection [26], disappears in 50% of infected animals after 45 days, and are absent in almost 100% of infected animals by 120 days post infection [27]. In the Morogoro district, the Mikese ward had the highest RVFV IgM seroprevalence followed by Magadu and Mazimbu, indicating that RVFV infections have recently occurred in the region and especially in the Mikese ward with 14 RVFV IgM positive cattle out of 38 analyzed. Other studies have also detected RVFV activity in cattle and humans in areas where the disease has never been reported before [12,15,19,28–30].

To summarize, we detected RVFV IgM antibodies in all study wards except Kajunjumele, with Morogoro district having a relatively high IgM seroprevalence compared to Kyela. These findings indicated the presence of active RVFV infection at the time of sampling, during the dry season, at least in the wards examined. Despite the small number of wards and animals tested for IgM, these findings clearly demonstrated the circulation of RVFV during IE periods in non-outbreak areas. It is not clear why the circulating RVFV in these areas did not lead into clinical disease, and the possible mechanisms for the virus maintenance remain to be elucidated. However, possible explanations could be circulation of non-virulent strains of RVFV in these areas or misdiagnosis excluding RVF for other febrile conditions with similar clinical features of fever and abortions. A limitation of the present study was that we unfortunately did not attempt to isolate RVFV from the IgM-positive animals, due to biosafety issues. RVFV is classified as a biosafety level-3 agent and demands biosecurity measures not available during the study. Furthermore, we did not perform any RT-PCR analysis to detect virus RNA.

The relatively high RVFV general seroprevalence recorded among cattle in Kyela (32%) and Morogoro (27%) districts could in some part be attributed to the physical characteristics of the respective district. The study site in Kyela is a low-lying area, close to Lake Nyasa, with many swamps and rivers and is subjected to regular flooding during the rainy seasons [9]. The ecology of low altitude and proximity to perennial water bodies were found to be associated with RVFV seropositivity in ruminant herds in Senegal and Madagascar, as well as in humans in Gabon and Tanzania [9,31–33]. Such an ecology provides good breeding habitats for mosquito vectors involved in the transmission of the RVFV. Furthermore, a large part of the study site in Kyela is used for wetland paddy cultivation with frequent water logging, suitable for mosquito breeding. In the Kyela district, cattle are usually grazed by tethering in open grassland, communal grazing land, near the wetlands and paddy farms, thus increasing the risk of acquiring RVFV from mosquitoes. On the other hand, the Kyela district is characterized by the abundance of banana and cocoa plantations. One study in Ngorongoro district, Tanzania, trapped more *Aedes aegypti* (a vector for RVFV) in banana and maize farms than in other habitats [34]. Thus, the banana and cocoa plantations in Kyela could form additional breeding habitats for mosquitoes that may transmit the virus to the animals and thus the observed high seroprevalence in this area.

Other factors that may contribute to the observed high RVFV seroprevalence include activities that facilitate animal movements such as livestock trade, moving animals to areas with green pastures during the drought season, lending animals among the community members and payment of dowry. Animal movements from high-risk areas could introduce RVFV into naïve animals in new areas [16,35] Thus, it is important to carry out studies also in areas found to have high RVFV seroprevalence to better understand the role of animal movements in the dispersal of RVFV and/or its vectors. Such data will be essential for formulation of RVFV control strategies.

The observed RVFV transmission hotspots during the sampling period in Magadu and Mikese point to locally existing factors playing a major role in RVFV maintenance and transmission dynamics. Although, entomological surveys were not conducted, the existence of suitable mosquito breeding habitats was evident. The presence of water in farms throughout the year provides suitable habitats for the breeding of RVFV mosquito vectors. Persistent water in aquaculture ponds and waste lagoons close to animal bans and grazing fields at Magadu farm may serve as important breeding habitats for the *Aedes* mosquito species. The presence of old machinery like tractors, discarded combined harvesters, old automobile tires and water storage containers may serve as water holding places thus providing harbour and breeding habitats for mosquitoes and continuous low-level transmission of RVFV to vertebrate hosts.

Older cattle (>2 years old) were found to be at a higher risk of having RVFV antibodies than younger cattle (OR = 0.19, 95% CI (0.1–0.35)). These findings agree with reports from studies which found higher seroprevalence in older animals [14,36–38]. The exotic breeds and their crosses are more susceptible to RVFV infection than local breed which are resistant and well adapted to the environment (12,13). However, in this study indigenous zebu breed appeared more likely to be RVFV seropositive than crosses (OR = 2.5, 95% CI (1.57–4.05). Sampled crossbred animals were from dairy farms with frequent use of acaricides to control tick infestation. The acaricides use could possibly prevent cattle from mosquito bites as well (Mnyone, 2018, personal communication) thus reducing the RVFV transmission in these animals. No significant difference between sex or the type of holding was observed.

Seroepidemiological studies provides the antibody status in respective species in each area, for the period during which the surveillance was carried out. Thus, continuous surveillance of the antibody prevalence in susceptible species is therefore highly recommended in epizootic/endemic areas.

## Conclusion and recommendations

The results demonstrated widespread prevalence of RVFV antibody among cattle during an inter-epizootic period in regions without previously reported RVF outbreaks. Therefore, it is important for animal health officers in these areas to be aware of the current RVFV circulation so that preventive measures such as vaccination could be implemented. It would be interesting to perform further studies in other similar areas with no history of RVF outbreaks, since it is likely that undetected low-level RVFV is occurring in many places. This would help to identify and target RVF hot spots by control measures, aiming for prevention of RVFV transmission to animals and humans.

Future studies could for instance focus on a more comprehensive and inclusive surveillance to identify and characterize RVFV reservoirs and vectors during the IE periods. Longitudinal investigations leading to a better understanding of ongoing RVFV circulation will lead to a better understanding of the IE virus maintenance.

## Supporting information

S1 Table (DOCX)
