# Supplementary material for: Serological evidence of inter-epizootic/inter-epidemic circulation of Rift Valley fever virus in domestic cattle in Kyela and Morogoro, Tanzania
Source: PLoS Negl Trop Dis. 2018 Nov 12;12(11):e0006931. doi: 10.1371/journal.pntd.0006931 (PMC6258417; doi:10.1371/journal.pntd.0006931)
Supplement: S1 Table — (DOCX) [file pntd.0006931.s001.docx]

Supporting information

**S1 Table.** RVFV plaque reduction neutralization (PRNT) titres. The 80% PRNT endpoints are indicated for all samples (n=104) that were c-ELISA positive

| **SN** | **District** | **Ward** | **80% PRNT endpoints** | |
| --- | --- | --- | --- | --- |
| 1 | KYELA | Bujonde | 1:40 |  |
| 2 | KYELA | Bujonde | 1:2560 |  |
| 3 | KYELA | Bujonde | 1:40 |  |
| 4 | KYELA | Bujonde | 1:40 |  |
| 5 | KYELA | Bujonde | 1:160 |  |
| 6 | KYELA | Bujonde | 1:160 |  |
| 7 | KYELA | Bujonde | 1:40 |  |
| 8 | KYELA | Bujonde | 1:40 |  |
| 9 | KYELA | Bujonde | 1:160 |  |
| 10 | KYELA | Bujonde | 1:40 |  |
| 11 | KYELA | Bujonde | 1:10 |  |
| 12 | KYELA | Bujonde | 1:40 |  |
| 13 | KYELA | Bujonde | 1:40 |  |
| 14 | KYELA | Bujonde | 1:640 |  |
| 15 | KYELA | Bujonde | 1:640 |  |
| 16 | KYELA | Bujonde | 1:40 |  |
| 17 | KYELA | Bujonde | 1:10 |  |
| 18 | KYELA | Bujonde | 1:40 |  |
| 19 | KYELA | Bujonde | 1:10240 |  |
| 20 | KYELA | Bujonde | 1:40 |  |
| 21 | KYELA | Bujonde | 1:640 |  |
| 22 | KYELA | Bujonde | 1:160 |  |
| 23 | KYELA | Bujonde | 1:160 |  |
| 24 | KYELA | Bujonde | 1:40 |  |
| 25 | KYELA | Bujonde | 1:40 |  |
| 26 | KYELA | Kajunjumele | 1:160 |  |
| 27 | KYELA | Kajunjumele | 1:10 |  |
| 28 | KYELA | Kajunjumele | 1:40 |  |
| 29 | KYELA | Kajunjumele | 1:40 |  |
| 30 | KYELA | Kajunjumele | 1:160 |  |
| 31 | KYELA | Kajunjumele | 1:40 |  |
| 32 | KYELA | Kajunjumele | 1:40 |  |
| 33 | KYELA | Kajunjumele | 1:10 |  |
| 34 | KYELA | Kajunjumele | 1:640 |  |
| 35 | KYELA | Kajunjumele | 1:2560 |  |
| 36 | KYELA | Kajunjumele | 1:40 |  |
| 37 | KYELA | Kajunjumele | 1:40 |  |
| 38 | KYELA | Kajunjumele | 1:160 |  |
| 39 | KYELA | Kajunjumele | 1:10240 |  |
| 40 | KYELA | Kajunjumele | 1:160 |  |
| 41 | KYELA | Katumba Songwe | 1:40 |  |
| 42 | KYELA | Katumba Songwe | 1:640 |  |
| 43 | KYELA | Katumba Songwe | 1:40 |  |
| 44 | KYELA | Katumba Songwe | 1:10 |  |
| 45 | KYELA | Katumba Songwe | 1:640 |  |
| 46 | KYELA | Katumba Songwe | 1:40 |  |
| 47 | KYELA | Katumba Songwe | 1:160 |  |
| 48 | MOROGORO | Magadu | 1:40 |  |
| 49 | MOROGORO | Magadu | 1:40 |  |
| 50 | MOROGORO | Magadu | 1:40 |  |
| 51 | MOROGORO | Magadu | 1:160 |  |
| 52 | MOROGORO | Magadu | 1:2560 |  |
| 53 | MOROGORO | Magadu | 1:160 |  |
| 54 | MOROGORO | Magadu | 1:160 |  |
| 55 | MOROGORO | Magadu | 1:640 |  |
| 56 | MOROGORO | Magadu | 1:640 |  |
| 57 | MOROGORO | Magadu | 1:40 |  |
| 58 | MOROGORO | Magadu | <10 |  |
| 59 | MOROGORO | Magadu | <10 |  |
| 60 | MOROGORO | Magadu | 1:2560 |  |
| 61 | MOROGORO | Magadu | 1:40 |  |
| 62 | MOROGORO | Magadu | 1:640 |  |
| 63 | MOROGORO | Magadu | <10 |  |
| 64 | MOROGORO | Magadu | 1:40 |  |
| 65 | MOROGORO | Magadu | 1:160 |  |
| 66 | MOROGORO | Magadu | 1:160 |  |
| 67 | MOROGORO | Magadu | 1:160 |  |
| 68 | MOROGORO | Magadu | 1:40 |  |
| 69 | MOROGORO | Magadu | 1:40 |  |
| 70 | MOROGORO | Magadu | 1:40 |  |
| 71 | MOROGORO | Magadu | <10 |  |
| 72 | MOROGORO | Magadu | 1:10 |  |
| 73 | MOROGORO | Magadu | 1:160 |  |
| 74 | MOROGORO | Magadu | 1:40 |  |
| 75 | MOROGORO | Magadu | 1:40 |  |
| 76 | MOROGORO | Magadu | 1:160 |  |
| 77 | MOROGORO | Magadu | 1:160 |  |
| 78 | MOROGORO | Magadu | <10 |  |
| 79 | MOROGORO | Magadu | 1:40 |  |
| 80 | MOROGORO | Magadu | 1:40 |  |
| 81 | MOROGORO | Mazimbu | <10 |  |
| 82 | MOROGORO | Mazimbu | <10 |  |
| 83 | MOROGORO | Mazimbu | <10 |  |
| 84 | MOROGORO | Mikese | 1:2560 |  |
| 85 | MOROGORO | Mikese | 1:40 |  |
| 86 | MOROGORO | Mikese | 1:40 |  |
| 87 | MOROGORO | Mikese | 1:640 |  |
| 88 | MOROGORO | Mikese | 1:640 |  |
| 89 | MOROGORO | Mikese | 1:10240 |  |
| 90 | MOROGORO | Mikese | 1:2560 |  |
| 91 | MOROGORO | Mikese | <10 |  |
| 92 | MOROGORO | Mikese | <10 |  |
| 93 | MOROGORO | Mikese | 1:640 |  |
| 94 | MOROGORO | Mikese | 1:160 |  |
| 95 | MOROGORO | Mikese | 1:10 |  |
| 96 | MOROGORO | Mikese | 1:160 |  |
| 97 | MOROGORO | Mikese | 1:40 |  |
| 98 | MOROGORO | Mikese | 1:40 |  |
| 99 | MOROGORO | Mikese | 1:160 |  |
| 100 | MOROGORO | Mikese | 1:160 |  |
| 101 | MOROGORO | Mikese | 1:40 |  |
| 102 | MOROGORO | Mikese | <10 |  |
| 103 | MOROGORO | Mikese | 1:40 |  |
| 104 | MOROGORO | Mikese | 1:40 |  |
